# Paraneoplastic Confusion: A Case of Anti-Hu Encephalitis

**DOI:** 10.7759/cureus.9205

**Published:** 2020-07-15

**Authors:** Dimitrios Drekolias, Naga Vaishnavi Gadela, Jason Jacob

**Affiliations:** 1 Internal Medicine, University of Connecticut Health Center, Farmington, USA; 2 Internal Medicine, University of Connecticut, Farmington, USA; 3 Internal Medicine, Hartford Hospital, Hartford, USA

**Keywords:** small-cell lung carcinoma, paraneoplastic syndromes, anti-hu encephalitis

## Abstract

Paraneoplastic manifestations are frequently seen in patients with small cell lung carcinoma (SCLC) and can present as diverse clinical entities ranging from endocrinopathies to neurological conditions. Anti-Hu encephalitis is a rare paraneoplastic manifestation most commonly seen in patients with SCLC. This case highlights an SCLC patient who presented with behavioral changes, cognitive deficits, and memory issues, and was found to have anti-Hu encephalitis. The subacute course of this clinical entity should be kept in mind and prompt further investigation in SCLC patients with these symptoms, especially when the laboratory workup of the major culprits is negative or inconclusive.

## Introduction

Small cell lung carcinoma (SCLC) is a subtype of lung cancer occurring almost exclusively in patients with smoking history. Its incidence compared to other subtypes of lung carcinoma has been steadily decreasing with the latest data from the Surveillance, Epidemiology, and End Results (SEER) indicating that SCLC comprises 13% of all lung cancers [1]. SCLC is frequently categorized as a primary lung malignancy of neuroendocrine origin along with the large cell neuroendocrine carcinoma and the carcinoid tumors of the lung, which appear to share a common genomic and proteomic profile [2-4]. SCLC may rarely lead to paraneoplastic syndrome involving the central and peripheral nervous system with the most common manifestations being the Lambert-Eaton myasthenic syndrome, sensory neuropathy, and limbic encephalitis. The neurological paraneoplastic syndrome of SCLC occurs up to 9% of SCLC cases [5]. More specifically, anti-Hu paraneoplastic disease can vary in presentation, exhibiting features of encephalitis, myelitis, or combined encephalomyelitis. Various sites of the central and peripheral nervous system can be affected, including the temporal lobes, the brainstem, the cerebellum, and the dorsal roots. We present a case of an SCLC-induced anti-Hu encephalitis. 

## Case presentation

**Figure 1:**
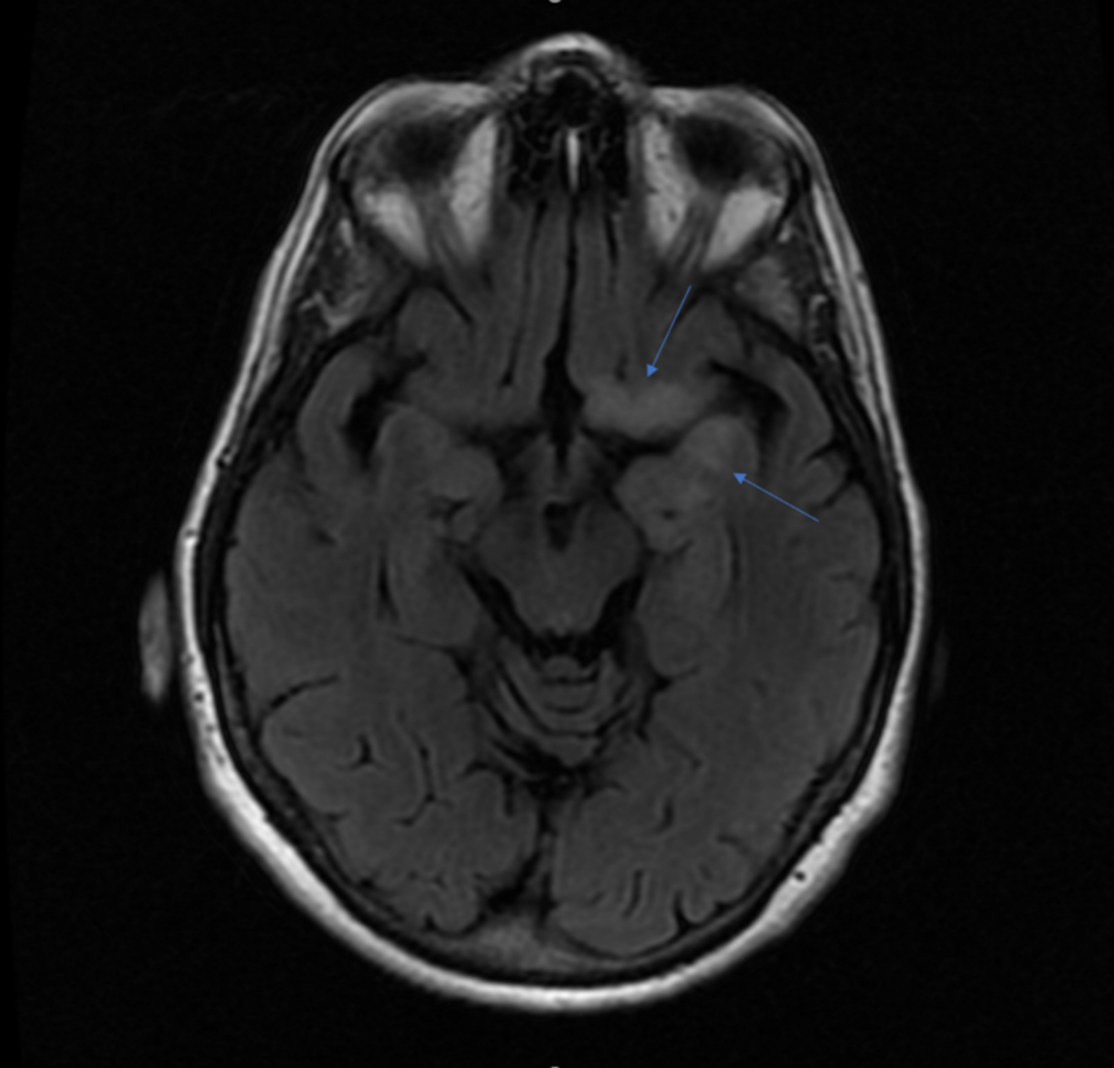
MRI of the brain revealing non-enhancing T2/fluid-attenuated inversion recovery (FLAIR) abnormalities affecting the area of the left frontal lobe, the left subcallosal region and the left mesial temporal lobe.

A 66-year-old female with a history of SCLC, paroxysmal atrial fibrillation on anticoagulation, chronic obstructive pulmonary disease, tobacco and alcohol use presented to the emergency department with progressively worsening altered mental status. The patient was noted to have changes in her behavior for the past two months prior to our initial encounter, which included confusion, short-term memory loss, and paranoid delusions. The patient had a history of alcohol abuse; however, she reported that she had not used alcohol for the past six months. She was diagnosed with SCLC approximately a year prior to this presentation and had completed chemotherapy regimen with carboplatin and etoposide. She was subsequently placed on atezolizumab maintenance therapy till approximately a month prior to this admission. Initial workup in the emergency department was notable only for a mild hyponatremia with a sodium of 133 mmol/L. The patient was afebrile and hemodynamically stable, and she was admitted for further workup of her encephalopathy. MRI of the brain was obtained and revealed non-enhancing T2/fluid-attenuated inversion recovery (FLAIR) abnormalities affecting the area of the left frontal lobe, the left subcallosal region and the left mesial temporal lobe (Figure 1).

The differential diagnosis had clinical entities such as infectious, autoimmune, paraneoplastic encephalitis, and malignancy. The patient subsequently underwent lumbar puncture which showed slightly elevated glucose of 79 mg/dL and abundant lymphocytes. Cerebrospinal fluid culture was negative. Oligoclonal bands and 14-3-3 were also absent. The patient was also tested extensively for infectious etiologies such as West Nile virus, syphilis, HIV, cytomegalovirus, John Cunningham (JC) virus, Borrelia burgdorferi, mycoplasma, and human herpesvirus 6, all of which returned negative. During the admission, the patient suffered a witnessed seizure. Based on the MRI results and the new-onset seizures, the decision was made to investigate paraneoplastic etiologies. An extensive paraneoplastic encephalitis panel comprising of several antibodies, including but not limited to anti-neuronal nuclear antibodies-1, -2, and -3 (ANNA-1, ANNA-2, ANNA-3), anti-alpha-amino-3-hydroxy-5-methyl-4-isoxazole propionic acid (anti-AMPA), and anti-N-methyl-D-aspartate (anti-NMDA), was ordered. The patient was also started on high-dose intravenous corticosteroids for a five-day course with significant improvement in terms of her mentation. Intravenous immunoglobulin was also administered for a five-day course after the improvement seen with the corticosteroid course. The patient was discharged with directions to follow up with the results of her paraneoplastic encephalitis panel. The panel demonstrated significantly elevated titers of the ANNA-1 (titer of 1:1,920) in the cerebrospinal fluid, which is also known as anti-Hu. In patients with SCLC, this is consistent with paraneoplastic encephalitis associated with anti-Hu. The clinical manifestations of behavioral changes, memory issues, and new-onset seizures, along with the MRI findings and the treatment response with corticosteroids and intravenous immunoglobulin, further support the diagnosis. 

## Discussion

SCLC can lead to a wide variety of paraneoplastic syndromes. Anti-Hu encephalitis is a paraneoplastic syndrome that is most commonly found in patients with SCLC [6]. There have been reports of anti-Hu encephalitis occurring in other types of cancer; however, 15% of the cases were free of any neoplasias [7]. Anti-Hu antibodies have also been observed in the pediatric population. In these cases, an association with the rare opsoclonus-myoclonus syndrome and with neuroblastoma was reported. Anti-Hu encephalomyelitis in children does not appear to be strongly associated with a malignancy. In one small cohort of eight children with anti-Hu encephalomyelitis, only two were reported to have cancer [8].

The anti-Hu paraneoplastic syndrome can be clinically diverse, presenting as encephalitis, myelitis, or combined encephalomyelitis. It can affect various anatomic sites of the central and peripheral nervous system, including the temporal lobes, the brainstem, the cerebellum, and the dorsal roots [9,10]. The clinical manifestations correlate with the anatomic sites involved with patients presenting with sensory neuronopathy when the dorsal roots are involved or with seizures, memory issues, or behavioral changes when the temporal lobes are involved [11,12].

Although rare, anti-Hu encephalitis should be kept in the differential diagnosis of SCLC patients who present with signs and symptoms of altered mental status, recent behavioral changes, seizures, or memory issues. The neurological outcome largely depends on timely treatment. In a large case series of 200 patients with anti-Hu encephalomyelitis, it was shown that prompt treatment of the underlying malignancy was associated with statistically significant increased likelihood of symptom amelioration and stabilization of neurological outcome (odds ratio of 4.6), as well as with a decreased mortality (relative risk was 2.6 compared with no treatment) [7]. Other treatment modalities associated with control of the symptoms include intravenous immunoglobulin, corticosteroids, plasma exchange, and human chorionic gonadotropin [10,13]. Another report of two patients with anti-Hu encephalomyelitis demonstrated improvement after administration of the anti-CD20 medication rituximab [14]. The prognosis is overall poor when vital structures such as the brainstem are involved; however, tumor regression, when achieved, has been associated with prolonged survival [15].

In our case, the extensive workup to investigate the underlying etiology of patient’s encephalopathic status was negative. The patient had a history of alcohol abuse; however, there were no signs of alcohol withdrawal or Wernicke encephalopathy and the patient’s supporting environment had reported that the patient had abstained from alcohol for approximately six months. The possibility of autoimmune encephalitis secondary to atezolizumab was also investigated; however, the patient had tolerated 11 cycles of maintenance therapy with this medication with no acute changes and the medication was discontinued about a month prior to admission. The rare encephalitis caused by atezolizumab has been reported to occur days after initiation of treatment, and it has been seen more frequently in patients with non-SCLC [16,17]. Given the history of the SCLC, which is frequently associated with paraneoplastic syndromes, the clinical rationale moved towards the exploration of paraneoplastic encephalitis. The positivity of the anti-Hu antibodies, in combination with the clinical manifestations of behavioral changes, memory issues, and new-onset seizures, was evidence for the case of anti-Hu encephalitis. The MRI findings and the treatment response to the corticosteroids and the intravenous immunoglobulin further supported our diagnosis.

## Conclusions

Anti-Hu encephalitis is a rare paraneoplastic manifestation most commonly seen in patients with SCLC. A high index of suspicion should be kept in SCLC patients with otherwise unexplained changes in behavior, memory issues, and new-onset seizures, especially when the laboratory workup of the major common culprits is negative or inconclusive. The timely identification of the disease is essential given the fact that prompt initiation of anti-tumor treatment could potentially lead to improvement of patient’s neurological status or stability of symptoms.
